# Efficient Dye-Sensitized Solar Cells Using Red Turnip and Purple Wild Sicilian Prickly Pear Fruits

**DOI:** 10.3390/ijms11010254

**Published:** 2010-01-20

**Authors:** Giuseppe Calogero, Gaetano Di Marco, Silvia Cazzanti, Stefano Caramori, Roberto Argazzi, Aldo Di Carlo, Carlo Alberto Bignozzi

**Affiliations:** 1 CNR, Istituto per i Processi Chimico-Fisici, Sede di Messina, Salita Sperone, C. da Papardo, I-98158 Faro Superiore Messina, Italy; 2 Dipartimento di Chimica, Università di Ferrara, Via L. Borsari 46, 44100 Ferrara, Italy; 3 c/o Dipartimento di Chimica, Istituto per la Sintesi Organica e la Fotoreattività (ISOF-CNR), Università di Ferrara, Via L. Borsari 46, 44100 Ferrara, Italy; 4 Dipartimento di Ingegneria Elettronica, Università di Roma 2, Tor Vergata, Roma, Italy

**Keywords:** solar cells, betalaine, opuntia, red turnip, natural dyes, solar energy

## Abstract

Dye-sensitized solar cells (DSSCs) were assembled by using the bougainvillea flowers, red turnip and the purple wild Sicilian prickly pear fruit juice extracts as natural sensitizers of TiO_2_ films. The yellow orange indicaxanthin and the red purple betacyanins are the main components in the cocktail of natural dyes obtained from these natural products. The best overall solar energy conversion efficiency of 1.7% was obtained, under AM 1.5 irradiation, with the red turnip extract, that showed a remarkable current density (Jsc = 9.5 mA/cm^2^) and a high IPCE value (65% at λ = 470 nm). Also the purple extract of the wild Sicilian prickly pear fruit showed interesting performances, with a Jsc of 9.4 mA/cm^2^, corresponding to a solar to electrical power conversion of 1.26%.

## 1. Introduction

Dye-sensitized solar cells (DSSCs) are devices for the conversion of visible light into electricity based on the photosensitization of wide band-gap metal oxide semiconductors [1–3]. Usually, the photoanode is prepared by adsorbing a dye (S) on a porous TiO_2_ layer. By this approach, the dye extends the spectral sensitivity of the photoelectrode, enabling the collection of lower energy photons. Due to its crucial role in such systems, considerable efforts have been directed towards the development and improvement of new families of organic dyes [4,5] and of metal complexes, the most efficient so far being Ru(II) [6–8] and Os(II) [9,10] polypyridine dyes, which couple broad spectral sensitivity to almost ideal ground and excited state thermodynamic and kinetic properties. Since the preparation of synthetic dyes normally requires multistep procedures, organic solvents and, in most cases, time consuming chromatographic purification procedures, there is interest towards the possible use of natural dyes which can be easily extracted from fruits, vegetable and flowers with minimal chemical procedures [11–13].

Natural pigments containing anthocyanins [14–19] and carotenoids [20] have shown overall solar energy conversion efficiencies below 1%. Betalain pigments represent an additional class of dyes of potential interest and purified extracts from commercial sources have been subjected to a detailed photoelectrochemical study [21]. These pigments are present in Caryophyllales plants, have high molar extinction coefficients in the visible region and pH dependent redox properties. The pigments are present in the different part of the plant including flowers petals, fruits, leaves, stems and roots. We report here the results of a series of experiments carried out on raw extracts of the following species: red turnip (*Beta vulgaris rubra, Kogel*), wild purple Sicilian prickly pear (*Opuntia engelmannii var. Lindhemeir)*, Sicilian indian fig (*Opuntia ficus indica, [L] Mill.*) and bougainvillea flowers.

**Figure 1.**
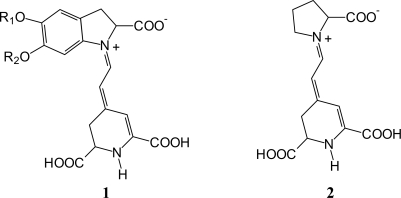
General structures of the main betalain dyes contained in the studied extracts: betacyanin (**1**) and indicaxanthin (**2**). R_1_ and R_2_ = H (betanidin) or R_1_ = β-d-glucose and R_2_ = H (betanin).

The red turnip originally grew wild in the Mediterranean area, particularly in regions that have cold nights during the spring season. Its ball-shaped red roots contain a high concentration of betalain pigments. The wild purple Sicilian prickly pear and the Sicilian Indian fig are both members of the Cactaceae family, originating from Mexico and widely distributed in much of Latin America, South Africa and in the Mediterranean area. Sicily ranks second among all countries in the world for producing and exporting wild Sicilian prickly pear fruits and Sicilian Indian figs. Bougainvillea plants are often grown in mild climates and typically have small flowers enclosed by large, brilliant red or purple *bracts* (modified leaves). The prevailing pigment coloration of the cited plants varies form orange to red, due to the combination of two main dyes: betacyanin (red-purple) and indicaxanthin (yellow-orange) whose schematic structures are reported in Figure 1 [22]. As shown in the figure, both dyes contain carboxylic functions which facilitate TiO_2_ surface binding.

## 2. Experimental

### 2.1. Preparation of Dye-Sensitizer Solutions

The synthetic dye [Ru(II)(2,2′-bipyridyl-4,4′-dicarboxylic-acid) (2,2′-bipyridyl-4,4′-ditetrabutyl-ammonium-carboxylate) (NCS)_2_], called N719, was synthesized and purified following the procedure reported in the literature [23]. N719 standard solution was prepared by dissolving 20 mg of the complex in 50 mL of ethanol. The fresh *Opuntia* fruits and the *Bougainvillea* flowers were harvested in Sicily, while the red turnip was taken from northern Italy. *Opuntia* fruits juices were principally prepared by crushing and squeezing the fresh fruits; the as prepared juices were filtered to remove solid fragments and stabilized at pH = 1.0 by addition of aqueous HCl or, alternatively by adding ascorbic acid until a final pH of 2.0 is reached. The red turnip and the bougainvillea extracts were obtained by immersing overnight red turnip slices and bougainvillea flowers’ leaves in HCl solution (0.1 M) respectively; the resulting extracts were centrifuged to remove any solid residue and used as such. Intentionally, any further purification of the extracts was avoided to check whether an efficient sensitization could be achieved with minimal chemical procedures. If properly stored, protected from direct sunlight and refrigerated at about +4 °C, the acidic natural dye solutions (pH = 5.0) are usually stable, with a deactivation half-time of more than 12 months [24].

### 2.2. Preparation of Electrodes

**Scheme 1.**
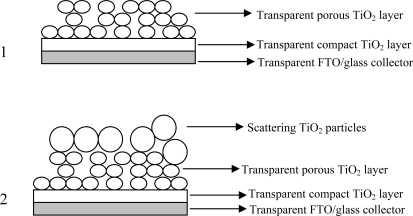
Cross sectional view of type A photoanodes without (1) and with (2) TiO_2_ scattering overlayer.

The conductive glass plates (FTO glass, fluorine-doped SnO_2_, sheet resistance 15 Ω/cm^2^) and the titanium oxide (TiO_2_) nanopowder (20 nm) were purchased respectively from Solaronix SA and Aldrich. Solvents and chemicals were of reagent or spectrophotometric grade and were used as received. The photoanodes were prepared by depositing TiO_2_ film on the FTO conducting glass: two edges of the FTO glass plate were covered with a layer of adhesive tape (3M Magic) to control the thickness of the film and to mask electric contact strips; successively the TiO_2_ paste was spread uniformly on the substrate by sliding a glass rod along the tape spacer. Two methods of preparation of colloidal TiO_2_ dispersion were employed (Scheme 1): according to method A, the semiconductor paste was prepared by blending 2.5 g of commercial TiO_2_ nanopowder (Aldrich), 4 mL of 0.1 M nitric acid, 0.08 g of polyethylene glycol (MW 8,000) and 0.2 mL of Triton X100 (Aldrich). The resulting suspension was stirred for 2 h and subsequently ultra-sonicated for additional 2 h; the resulting mesoscopic oxide film was around 8–10 μm thick and opaque. In Method B a sol-gel procedure for preparing titanium oxide nanoparticles described elsewhere [25] was followed; before depositing the colloidal TiO_2_ dispersion on the conducting glass a compact thin underlayer of TiO_2_ (blocking layer) was created onto the FTO surface by spin coating (3,000 rpm) of a 0.2 M titanium isopropoxide solution in ethanol, followed by firing at 450 C° for 30′. A final treatment of the porous TiO_2_ electrodes with aqueous TiCl_4_ [6] led to thin (5–6 μm) transparent multilayered electrodes. For control measurements, analogous electrodes were prepared without blocking layer. After drying the coated plates prepared according to the two different methods were sintered in air for 1 h at 450 °C. Furthermore, a third type of electrode equipped with a scattering layer was prepared by casting the TiO_2_ nanopowder (method A) on top of the transparent photoanodes obtained according to method B.

All the three types of photoanodes were immersed into the natural dye solutions, at room temperature for one night, rinsed with distilled water and ethanol and subsequently dried. N719 sensitized electrodes were only rinsed with ethanol. Pt coated counter electrodes were prepared according to published procedures [25].

### 2.3. DSSC Assembling

The solar cells were assembled according to the following procedures: the electrolyte solution (generally constituted by 0.7 M LiI and 0.07 M I_2_ in 3-methoxyproprionitrile) was poured on the mesoporous TiO_2_ film. The counter electrode was pressed against the electrolyte impregnated anode and clamped firmly in a sandwich configuration. Parafilm sealed cells (0.5 cm^2^ active area) were built by pressing the sensitized photoanode against the counter electrode equipped with a parafilm frame (120 μm) used to confine the liquid electrolyte inside the cell. In this configuration the cell was stable towards solvent evaporation and leaking for several days even using a volatile solvent like acetonitrile. Hermetically sealed cells were prepared to study the long-term stability under simulated solar light. In this case, the photoanode and the Pt counter electrode were sandwiched with a 60 μm thick (before melting) surlyn polymer foil as spacer. Sealing was done by keeping the structure in a hot-press at 100 °C for 15–30 seconds. The liquid electrolyte was introduced into the cell gap through a predrilled hole on the counter electrode. The hole was then covered with a glass disk sealed with a Surlyn layer.

### 2.4. Measurements

The absorption spectra were recorded on a Perkin-Elmer L20 spectrophotometer UV–Vis-NIR or on a JASCO V 570 UV-Vis-NIR spectrophotometer. For the IPCE spectra [7] the cell was illuminated with a water cooled Osram XBO 150W Xe lamp coupled with an Applied Photophysics high radiance monochromator (spectral bandwidth of 10nm). The irradiated area was 0.5 cm^2^. Photocurrents were measured under short circuit conditions with an Agilent 34401A digital multimeter. Incident irradiance was measured using a 1 cm^2^ Centronic OSD100-7Q calibrated silicon photodiode. Calorimetric measurements were carried out by using a Perkin Elmer Pyris 1 differential scanning calorimeter (DSC) calibrated with indium and zinc standards. Current-Voltage curves were recorded by a digital Keithley 236 multimeter connected to a PC and controlled by a homemade program. Simulated sunlight irradiation was provided by a LOT-Oriel solar simulator (Model LS0100-1000, 300 W Xe Arc lamp powered by LSN251 power supply equipped with AM 1.5 filter, 100 mW/cm^2^). Cell active area was 0.5 cm^2^. Incident irradiance was measured with an ORIEL radiant power meter equipped with an ORIEL thermopile detector. These measurements were cross checked with a different irradiation setup consisting of an air cooled HID (metal halides) lamp set at 0.1 W/cm^2^. In this case the current output of each cell was recorded by linearly varying the potential from 0 to 0.7 V in a two electrode configuration using a scan speed of 10 mV/s by employing an EcoChemie PGSTAT 30/2 electrochemical workstation interfaced with a personal computer. The results obtained with two experimental configurations were substantially coincident.

## 3. Results and Discussion

### 3.1. Absorption Spectra of Raw Natural Dye Extract

**Figure 2.**
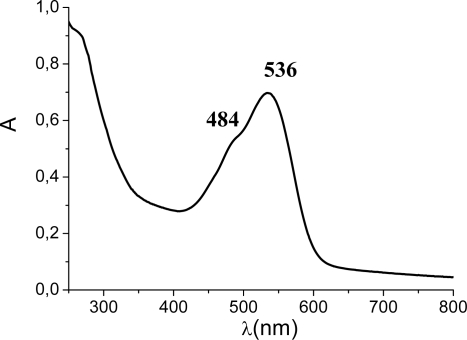
UV-Vis spectrum of raw red turnip extracts in 0.1 M HCl solution showing the betaxanthin (**2**) (484 nm) and betanin (**1**) (536 nm) visible absorption.

Betalain extracted from red-turnip in 0.1 M HCl solution displayed an intense absorption in the 400–600 nm region due to the mixed contributions of the yellow-orange betaxanthins (480 nm) and of the red-purple betacyanines (540 nm) (Figure 2). These absorptions originate from π–π^*^ transitions and DFT calculations carried out on betanidin [26] have pointed out their essential charge transfer character, with the LUMO centered on the dihydropyridine portion of the molecule. Compared to neutral extracts, dye cocktails extracted in acidic conditions present a stronger absorption contribution at lower wavelengths, indicating an increase of the indicaxanthin concentration. This is consistent with the fact that indicaxanthin is mainly contained in vacuoles whose membranes are lysed in acidic conditions.

**Figure 3.**
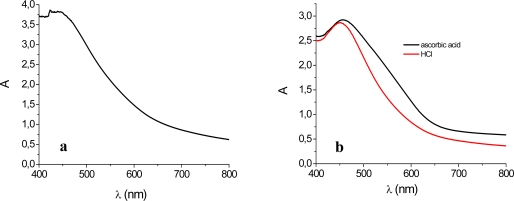
Absorption spectrum of transparent TiO_2_ film stained with acidic *Beta vulgaris rubra* (a) and *Opuntia engelmannii* extracts (b).

Upon adsorption on the TiO_2_ electrodes of both red turnip and wild Sicilian prickly pear fruits extracts, the visible absorption band shifts to higher energy, showing a broad maximum around 470–450 nm. This is most probably determined by a preferential binding of indicaxanthin (**2**). As previously observed by other authors [21], the acidic environment was essential for obtaining betalain sensitized photo-electrodes characterized by high optical densities, capable of an almost complete absorption of visible photons in the 400–600 nm range (Figure 3). The reason is ostensibly related to protonation of betalainic carboxylic groups which are otherwise unable, in their anionic form to bind to the TiO_2_ surface. The conditions of dye extraction have also repercussions on the light harvesting efficiency of the sensitized photoanodes. For example, in the case of the wild Sicilian prickly pear juices the absorption spectra of the anode prepared from the solution stabilized with ascorbic acid (Figure 3b, black line) presents a wider shoulder at longer wavelengths in comparison with the equivalent anode stabilized with HCl (Figure 3b, red line), indicating a relatively higher percentage of betanin adsorption. It must be noted that the red shift in Figure 3(b) cannot be originated by direct ascorbic acid adsorption, since the ascorbate-TiO_2_ charge transfer band is centered at 400 nm [27].

### 3.2. Photoelectrochemistry

**Figure 4.**
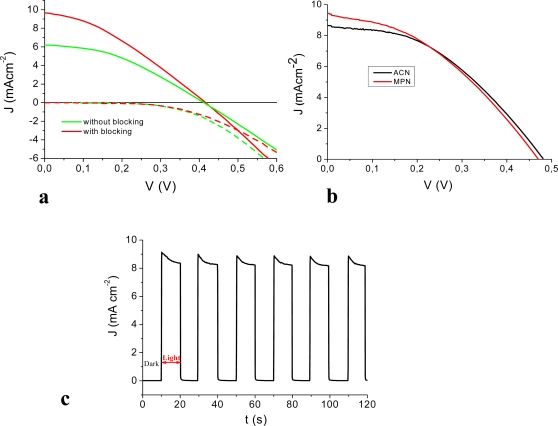
Photoelectrochemical performances obtained with a red turnip sensitized solar cell: (a) with (red line) and without (green line) blocking underlayer; (b) in the presence of an ACN (black line) and MPN/ionic liquid electrolyte (red line); (c) short circuit photocurrent transients in the presence of the MPN-ionic liquid electrolyte. Cells in (b) and (c) are equipped with a blocking underlayer.

Red turnip extracts displayed promising photoelectrochemical performances showing Jsc = 6 mA/cm^2^, Voc = 0.41 V, fill factor = 0.4, active area = 0.5 cm^2^ and η = 1% using an electrolyte composed of 0.5 M/0.05 M LiI/I_2_ in acetonitrile (ACN) (anode prepared with method B without blocking layer). The application of a compact TiO_2_ underlayer (*i.e.* blocking layer) was instrumental for enhancing the cell performance, increasing the short circuit photocurrent up to 9.4 mA/cm^2^ and the photovoltage to 0.48 V (Figure 4a). The use of a less volatile electrolyte, more suitable for practical applications, composed of 0.6 M propylmethylimidazolium iodide (PMII), 0.1 M LiI and 0.2 M I_2_ in methoxypropionitrile (MPN) left almost unchanged the J-V characteristic of the cell (Figure 4b), allowing to obtain overall efficiencies of the order of 1.75% (active area 0.5 cm^2^), *to our knowledge among the highest so far reported with raw natural dyes*. Chronoamperometry experiments under no potential bias in the presence of the PMII ionic liquid in MPN, showed rectangular shaped photocurrent transients indicating a good reproducibility of the cell response upon subsequent irradiation cycles (Figure 4c). It must be noted that under identical conditions and employing an anode prepared with method B, a N719 sensitized cell (active area = 0.5 cm^2^) generated Jsc = 17.73 mA/cm^2^, Voc = 0.53 V, and η = 3.3%. The presence of the compact underlayer seems to be essential for reducing the back recombination from the Fluorine doped Tin Oxide (FTO) electron collector. Indeed our findings agree with a recent paper by Burke *et al*. [28] in which it is pointed out that, compared to bulkier Ru(II) complexes, smaller and flat organic molecules like the sensitizers reported here may not be able to insulate well the underlying conductive oxide from the oxidized electrolyte (namely I_3_^−^) and that, for this reason, interface optimization is required to exploit the full potentialities of such dyes. The performances of the wild Sicilian prickly pear dyes were also very close to the red turnip, exhibiting a short circuit photocurrent close to 8 mA/cm^2^ which could be brought to about 10 mA/cm^2^ with the use of a scattering TiO_2_ overlayer (see Table I). On the contrary, Bougainvillea and Sicilian Indian fig were a poorer source of betalain dyes. Indeed cells fabricated with such raw extracts only achieved modest power conversion efficiencies, with maximum photocurrents slightly higher than 2 mA/cm^2^, due to a modest light harvesting capability (A_max_ = 0.3–0.5).

In general, natural dyes suffer from low Voc, which is at best 100 mV lower than an equivalent N719 sensitized cell. This can be due both to possible efficient electron/dye cation recombination pathways and to the acidic dye adsorption environment. In fact, it is well known that H^+^ are potential determining ions for TiO_2_ and that proton adsorption causes a positive shift of the Fermi level of the TiO_2_, thus limiting the maximum photovoltage that could be delivered by the cells. Attempts of increasing the photovoltage by addition of terbutyl pyridine were totally unsuccessful: considering the red turnip based solar cell, to a modest gain in photovoltage and in fill factor (0.53 V and 0.53 respectively) it corresponded to a dramatic drop in photocurrent (1.53 mA/cm^2^). This phenomenon, which has been independently verified in our two laboratories, is probably determined by an enhancement of the indicaxanthin reducing activity under basic conditions, [29] leading to dye degradation due to side reactions with other chemical species like oxidised electrolyte (I_3_^−^ or I_2_), dissolved oxygen and other minor impurities. This effect was not documented by Zhang *et al*. [20] which were using purified betanin, which, indeed, is more robust and does not readily decompose following pH changes. Nevertheless, pure betanin results a rather poor sensitizer, despite the extended spectral absorption at lower energy. Still, at present, the reason is not completely clear, but it has been pointed out [11] that often raw natural dye mixtures exhibit better performance than commercial or purified analogues. This could be related to the presence in the natural extract of specific pools of ancillary molecules (*i.e.,* alcohols, organic acids, *etc.*) which act as coadsorbates, suppressing recombination with the electrolyte, reducing dye aggregation and favouring charge injection.

**Figure 5.**
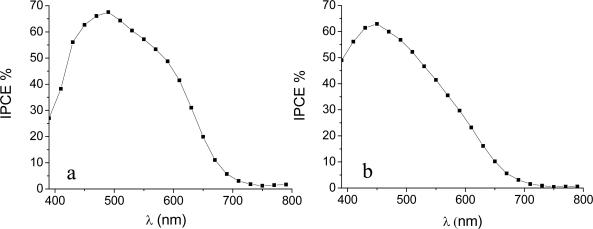
IPCE spectra of: (a) *Beta vulgaris rubra* and (b) *Opuntia engelmannii*. All experiments were performed with 0.5 M LiI/0.05M I_2_ electrolyte and type B photoanodes (see experimental section).

Photoaction spectra (Figure 5) provided further insights on the photoelectrochemical behaviour of this family of natural dyes. The photoaction spectra are in good agreement with the absorption spectrum of the sensitized TiO_2_ film, showing a maximum of 65% in the 450–470 nm region, testifying the charge injection from the excited state of the natural sensitizers and that no other species are significantly contributing to charge injection. Although some light scattering from the solid thin film complicate a precise evaluation of the true absorbance of the photoelectrode from the absorption spectra, by subtracting the longest wavelength background, maximum optical densities > 2 can be safely estimated. Thus photon absorption in the 400–600 nm interval is >99% complete and conversion efficiencies inferior to 80–85% (a value limited by the transmittance of the conductive glass) can only be determined by an injection efficiency and/or by an electron collection efficiency smaller than unity. Thus, the IPCE is essentially determined by the φ_inj_η product where φ_inj_ is the charge injection efficiency and η is the electron collection efficiency.

Spectroscopic and electrochemical investigations with raw dye solutions present some difficulties, related to the presence of a mixture of dyes, however we were able to provide a reasonable estimate of the ground and excited state energetics of the betalain sensitizers. Cyclic voltammetry of red turnip extracts at a glassy carbon electtode resulted in a broad irreversible peak centred at 0.75 V *vs.* SCE. The same electrochemical process could be observed by using an FTO electrode whose surface was functionalized with the same dye extract used for TiO_2_ adsorption. Thus, it could be reasonably assigned to the oxidation of the pool of betalain dyes which also adsorbs onto TiO_2_. By using E_peak_–E^00^, where E_peak_ is the peak potential of the irreversible oxidation wave of the dyes, and E^00^ is the optical excitation energy measured from the onset of absorption spectrum (Figure 2), an excited state oxidation potential (−1.3 ± 0.05 V *vs* SCE), negative enough to allow for a good energetic superimposition with the d band of the TiO_2_, was obtained. Electronic coupling with empty TiO_2_ states should also be good, since DFT calculations [27] showed a localization of the LUMO on the dihydropyridine ring of the betalain, the portion of the molecule which should be directly linked to the anatase surface. Based on these indications, injection into the conduction band of the semiconductor should be an activationless process, which is unlikely to limit the IPCE. On the other hand, recombination losses can reduce the η factor. The importance of a blocking layer in controlling recombination with the electrolyte has already been pointed out, but, in the case of natural dyes, also recombination with the oxidized dye can be relevant. The ground state oxidation potential of 0.7/0.75 V *vs.* SCE should ensure a good dye regeneration rate by iodide, whose E_1/2_ lies at about 0.4 V *vs.* SCE. However, compared to ruthenium sensitizers in which the hole is confined into a metal centred d orbital, relatively decoupled from the semiconductor surface, electron recapture by betalain dye cation is expected to be faster, since the hole is located in closer proximity to the nanoparticle surface. This is suggested by the behaviour of a related series of cyanine dyes showing on the same TiO_2_ substrate recombination lifetimes lower than 200 ns [30] and will be clarified in the near future by nanosecond laser flash photolysis measurements.

### 3.3. Stability Test

**Figure 6.**
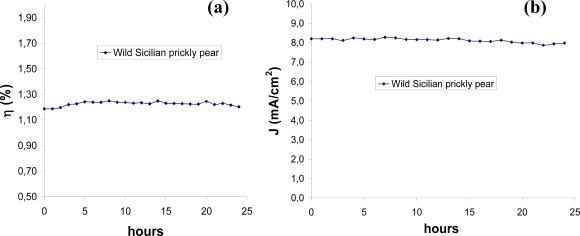
Variation of current-voltage parameters of wild Sicilian prickly pear based DSSCs. All experiments were carried out under 1 sun illumination (100 mW/cm^2^, air mass 1.5) with 0.5 M LiI/0.05M I_2_ electrolyte and type B photoanodes on a hermetically sealed solar cell (see experimental section). (a) Solar energy conversion efficiency (η). (b) Short circuit current density (Jsc).

Preliminary tests on the stability of these natural dyes were carried out by monitoring some indicative parameters, such as Jsc and η, under continuous AM 1.5 solar irradiation for 24 h, without any cooling system (the temperature of the cell reached 60–65 °C during the long term stability test) and no significant changes were observed, as reported in Figure 6.

**Figure 7.**
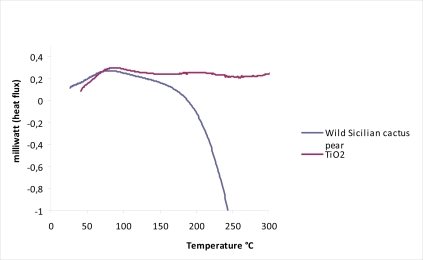
DSC curve for bare TiO_2_ (purple) and for betalain (*Opuntia engelmanni*) sensitized TiO_2_ (blue).

Besides photoelectrochemical stability, thermal stability was evaluated by calorimetric analysis. Thermograms were recorded on a 10 mg wild Sicilian prickly pear sensitized TiO_2_ sample, by using a heating rate of 10 °C/min in the 50–400 °C temperature range. All the thermograms were baseline subtracted and normalized to 1 mg of sample. The DSC curve (Figure 7) present thermal stability below 160 °C and a decomposition process starting at 180 °C. The weight loss at 230 °C was attributed to the decarboxylation. These results indicate that betalain dyes (adsorbed in TiO_2_ matrix) should be thermally stable at the temperatures reached by the operational cell under solar irradiation. Longer stability tests are still in progress in order to establish the life time of DSSCs based on betalains sensitizers.

## 4. Conclusions

In this work we have reported an investigation on betalain pigments as natural photosensitizers, describing and comparing their sensitization activity with respect to one of the best ruthenium dyes (N719). Preliminary stability tests also showed promise for practical applications. Betalain raw pigments simply extracted in acidic conditions from vegetable and fruits achieved IPCEs higher than 60% and solar energy conversion efficiency of 1.7%, about half of that obtained with an equivalent N719 sensitized cell. A notable increase in cell performance was observed by using a TiO_2_ blocking underlayer which partly suppresses charge recombination with the oxidized electrolyte. Natural dye based cells appear to be limited by low Voc, which can be slightly improved, as usual, by addition of ter-butyl-pyridine, but at the cost of a large decrease in photocurrent, probably due to indicaxanthin degradation. Finding different additives for improving Voc might result in larger conversion efficiencies. Although the efficiencies obtained with these natural dyes are still below the current requirements for large scale practical application, the results are encouraging and may boost additional studies oriented to the search of new natural sensitizers and to the optimization of solar cell components compatible with such dyes.

## Figures and Tables

**Table 1.** Photovoltaic performances with betalain dyes from different sources (* indicates pH = 1.0).

**Dye Source**	**Jsc (mA/cm)**	**Voc (mV)**	**FF**	**η (%)**	**Anode Type (active area 0.5 cm^2^)**
N719	17.73	530	0.35	3.3	Method B
Red Turnip* (*Beta vulgaris rubra*)	9.5	425	0.37	1.7	Method B
Wild Sicilian Prickly pear* (*Opuntia engelmannii*)	9.4	350	0.38	1.26	Scattering layer
Wild Sicilian Prickly pear* (*Opuntia engelmannii*)	8.20	375	0.38	1.19	Method B
Wild Sicilian Prickly pear* (*Opuntia engelmannii*)	7.32	400	0.41	1.21	Method A
Sicilian Indian Fig* (*Opuntia ficus indica*)	2.7	375	0.54	0.50	Method A
Bougainvillea*	2.1	300	0.57	0.36	Method A
